# Tumor-specific expression of αvβ3 integrin promotes spontaneous metastasis of breast cancer to bone

**DOI:** 10.1186/bcr1398

**Published:** 2006-04-11

**Authors:** Erica K Sloan, Normand Pouliot, Kym L Stanley, Jenny Chia, Jane M Moseley, Daphne K Hards, Robin L Anderson

**Affiliations:** 1 Peter MacCallum Cancer Centre, Melbourne, Victoria, Australia; 2 St Vincent's Institute of Medical Research, Fitzroy, Victoria, Australia; 3 Present Address: Division of Hematology-Oncology, UCLA School of Medicine, Los Angeles, California, USA

## Abstract

**Introduction:**

Studies in xenograft models and experimental models of metastasis have implicated several β3 integrin-expressing cell populations, including endothelium, platelets and osteoclasts, in breast tumor progression. Since orthotopic human xenograft models of breast cancer are poorly metastatic to bone and experimental models bypass the formation of a primary tumor, however, the precise contribution of tumor-specific αvβ3 to the spontaneous metastasis of breast tumors from the mammary gland to bone remains unclear.

**Methods:**

We used a syngeneic orthotopic model of spontaneous breast cancer metastasis to test whether exogenous expression of αvβ3 in a mammary carcinoma line (66cl4) that metastasizes to the lung, but not to bone, was sufficient to promote its spontaneous metastasis to bone from the mammary gland. The tumor burden in the spine and the lung following inoculation of αvβ3-expressing 66cl4 (66cl4beta3) tumor cells or control 66cl4pBabe into the mammary gland was analyzed by real-time quantitative PCR. The ability of these cells to grow and form osteolytic lesions in bone was determined by histology and tartrate-resistant acid phosphatase staining of bone sections following intratibial injection of tumor cells. The adhesive, migratory and invasive properties of 66cl4pBabe and 66cl4beta3 cells were evaluated in standard *in vitro *assays.

**Results:**

The 66cl4beta3 tumors showed a 20-fold increase in metastatic burden in the spine compared with 66cl4pBabe. A similar trend in lung metastasis was observed. αvβ3 did not increase the proliferation of 66cl4 cells *in vitro *or in the mammary gland *in vivo*. Similarly, αvβ3 is not required for the proliferation of 66cl4 cells in bone as both 66cl4pBabe and 66cl4beta3 proliferated to the same extent when injected directly into the tibia. 66cl4beta3 tumor growth in the tibia, however, increased osteoclast recruitment and bone resorption compared with 66cl4 tumors. Moreover, αvβ3 increased 66cl4 tumor cell adhesion and αvβ3-dependent haptotactic migration towards bone matrix proteins, as well as their chemotactic response to bone-derived soluble factors *in vitro*.

**Conclusion:**

These results demonstrate for the first time that tumor-specific αvβ3 contributes to spontaneous metastasis of breast tumors to bone and suggest a critical role for this receptor in mediating chemotactic and haptotactic migration towards bone factors.

## Introduction

Breast cancer affects one in ten women in developed nations. The prognosis is favorable for women with clinically confined tumors at the time of diagnosis, but mortality rates are greater than 80% in cases where the tumor has metastasized to distant sites [1]. Metastasis to bone occurs frequently in advanced breast cancer and is accompanied by debilitating skeletal complications [2]. Current treatments are largely palliative and new therapies that specifically prevent the spread of breast cancer to bone are urgently required. Little is known, however, about the molecular determinants that regulate homing of breast cancer cells to bone.

Integrins are dimeric adhesion receptors that mediate cellular attachment to the extracellular matrix (ECM) or to adjacent cells. Interaction of integrins with their substrates regulates various cellular functions associated with tumor development and metastatic progression, including cell adhesion, migration, invasion, proliferation and survival/anoikis [3]. The changes in the integrin activation state and the alteration in the level of expression of integrins or their ECM ligands have therefore been extensively documented and are thought to contribute to neoplastic progression [4-6].

Studies examining the expression of αvβ3 integrin in various tumor tissues have been strongly suggestive of a potential role for this receptor in tumor progression, particularly for invasive tumors that preferentially metastasize to bone, such as breast and prostate carcinomas [7,8]. β3-type integrins (αvβ3 and α_IIb_β3) are expressed in multiple cell types including invasive tumor cells, osteoclasts, activated endothelial and smooth muscle cells, platelets, megakaryocytes and macrophages [9]. Accordingly, the contribution of several of these β3-expressing cell populations to tumor growth and metastatic progression has been demonstrated in studies using specific inhibitors and/or genetic ablation of β3 receptors in animal models [10-15].

Consistent with enhanced endothelial expression of αvβ3 integrin in the tumor vasculature [10,11,16,17] and its role in promoting primary tumor growth, small molecule antagonists or function-blocking antibodies targeting β3 integrins exert anti-tumor effects concomitant with decreased tumor vascularization in melanoma, prostate cancer and breast cancer xenograft models [11,12,18-20]. Interestingly, studies examining tumor growth in mice null for β3 integrin have yielded results apparently at odds with the inhibitor data. In contrast to the decreased tumor growth and angiogenesis observed upon treatment with β3 inhibitors, these responses are enhanced in β3-null mice [13,21]. To reconcile these studies, it was proposed that αvβ3 may function as an angiogenic switch, promoting vessel growth when ligated but triggering an apoptotic response in αvβ3-expressing endothelial cells in the absence of appropriate ligand. In β3-null mice the absence of endothelial αvβ3 may remove apoptotic signals, allowing excessive endothelial growth [21,22].

Some of the discrepancies between studies using β3 antagonists and genetic ablation of β3 may be attributed also in part to the lack of receptor specificity for some of the inhibitors used *in vivo *or to their direct effect on tumor cells, as previously suggested [22]. β3 inhibitors would presumably inhibit both host populations and tumor cell populations expressing these receptors, whereas β3 ablation would target only host populations, including cells of the innate immune system, which may exert either pro-tumor or anti-tumor effects and produce a net stimulatory effect on primary tumor growth [13].

While there is strong correlative evidence supporting the role of tumor-specific αvβ3 expression in breast cancer progression [7,23,24], its precise contribution to primary tumor growth and metastasis is still unclear. Human breast tumor lines expressing αvβ3 are typically more metastatic in xenograft models than those that do not express this receptor [25]. From *in vitro *studies, a variety of functions likely to contribute to tumor progression have been ascribed to αvβ3 expressed on tumor cells. In particular, αvβ3 mediates breast tumor cell adhesion to vitronectin and other bone ECMs and promotes their survival, migration and invasion on bone matrix *in vitro *[25-29]. These processes can be inhibited by anti-αvβ3 antibodies or antagonists [25,27,28,30]. In addition, activated αvβ3 regulates protease maturation, is required for breast tumor cell interaction with platelets [5,14,31] and contributes to breast tumor cell adhesion to the subendothelial matrix under dynamic blood flow conditions [32].

*In vivo *studies addressing specifically the role of tumor cell αvβ3 on primary tumor growth are conflicting, however. Exogenous expression of wildtype or constitutively active αvβ3 in transformed human astrocytes exerts suppressive effects on intracranial growth of gliomas, giving rise to fewer and smaller tumors [33]. Expression of αvβ3 in 21NT human mammary carcinoma cells is insufficient alone to enhance their growth in the mammary fat pad of nude mice [34], whereas the same approach reduces subcutaneous growth of MCF-7 cells in nude mice [35]. It is also probable that tumor αvβ3 may have different roles at various stages of metastatic progression. In fact, expression of activated αvβ3 in melanoma and breast cancer cells enhances experimental metastasis to the lung following intravenous injection of tumor cells (which bypasses the formation of a primary tumor) in immune-compromised mice [14,36]. Enhanced metastasis of breast tumor cells was postulated to be mediated through tumor-induced platelet aggregation and arrest in capillaries as these processes are inhibited by antibodies targeting specifically platelet α_IIb_β3 or human αvβ3 on tumor cells [14]. Whether these interactions are required for spontaneous metastasis of breast tumors from orthotopic sites remains unclear, however.

Vascular or oral administration of selective inhibitors of αvβ3 S247 and S137, respectively, was reported to significantly reduce spontaneous metastasis of 435/HAL breast tumor cells to the lung from the mammary gland in severe combined immunodeficient mice [37]. Neither drug inhibited primary tumor growth or platelet aggregation at the concentration used, and thus the antimetastatic effects are unlikely to be mediated through these processes. Similar evidence supporting the role of tumor-specific αvβ3 in bone metastasis has been reported. Chinese hamster ovary cells or MDA-MB-231 variants overexpressing αvβ3 are more metastatic to bone than their respective parental line when inoculated intravenously [38]. Conversely, treatment of mice with the selective αvβ3 inhibitor S247 dramatically reduces the incidence and size of MDA-MB-435 osteolytic lesions whereas the specific platelet aggregation inhibitor ML464 prevents B16 melanoma metastases to bone in the intracardiac experimental model [15,39].

While informative, the lack of a primary tumor in experimental models of bone metastasis makes it difficult to assess whether tumor αvβ3 is required at the primary site, the metastatic site or both sites for spontaneous metastasis from the mammary gland to bone. Conversely, current xenograft models of breast cancer metastasis are poorly metastatic to bone from the orthotopic site and thus have not been used to investigate the role of tumor αvβ3 in spontaneous breast cancer metastasis to bone. To circumvent these problems, we have used a syngeneic orthotopic model of spontaneous breast cancer metastasis developed in our laboratory [40,41] and asked whether exogenous expression of αvβ3 integrin in 66cl4 mammary carcinoma cells that metastasize to the lung but not bone is sufficient to promote their spontaneous metastasis from the mammary gland to bone. The model allows the simultaneous measurement of the impact of αvβ3 expression on primary tumor growth and on the metastatic burden in bone in immunocompetent Balb/c mice. Our results indicate that tumor expression of αvβ3 does not alter the proliferation of 66cl4 cells *in vitro *or in the mammary gland, and nor is it required for their growth in bone. The expression of αvβ3 in these cells, however, is sufficient to promote their spontaneous metastasis to bone. Assays mimicking various steps of the metastatic process suggest a critical role for this receptor in regulating the chemotactic response of mammary carcinoma cells to bone-derived factors, in regulating adhesion and migration towards bone matrix proteins and in the recruitment of osteoclasts to bone metastatic sites.

## Materials and methods

### Cell and cell culture

The mouse mammary epithelial cell lines 4T1, 66cl4 and 67NR were derived by Dr F Miller (Michigan Cancer Foundation, Detroit, MI, USA) [42]. 4T1.2 and 4T1.13 are clonal cell lines derived from 4T1 by our laboratory [40,41]. These cell lines were cultured in alpha minimal essential medium (α-MEM) supplemented with 5% FCS and 1% penicillin-streptomycin, at 37°C, 5% CO_2_. The ecotropic packaging cell line Phoenix was a gift from Dr G Nolan (Stanford University, CA, USA) and was cultured in DMEM supplemented with 10% FCS and antibiotics at 37°C, 5% CO_2_.

The murine microvascular endothelial cell line bEnd.3 was kindly provided by Dr R Hallman (Jubileum Institute, Sweden) and was maintained in DMEM supplemented with 10% FCS, glutamine (2 mM), glucose (4.5 mg/ml) and 1% penicillin-streptomycin.

### Generation and analysis of 66cl4beta3 cells

cDNA encoding full-length mouse β3 integrin (a generous gift from Dr S Teitelbaum, Washington University, St Louis, MO, USA) was subcloned into pBabe-puro retroviral vector [43]. Phoenix cells were transiently transfected with target cDNA and culture supernatant was used to infect 66cl4 cells. Stably infected cell lines were selected by treatment with 9 μg/ml puromycin over 7 days. Detection of cell-surface integrin expression was performed by standard flow cytometry. Briefly, the cells (1 × 10^6^) were resuspended in blocking buffer (α-MEM supplemented with 2% BSA and 2% FCS) for 30 minutes on ice. The cells were then incubated with 2 μg/ml hamster anti-mouse-αv, anti-β3 or isotype control primary antibody (BD Pharmingen, North Ryde, NSW, Australia) diluted in labeling buffer (α-MEM supplemented with 2% FCS) for 1 hour on ice. Unbound antibodies were removed by washing twice with PBS, 2% FCS, and the cells were treated with a fluorescein isothiocyanate-conjugated mouse anti-hamster secondary antibody cocktail (Pharmingen) in labeling buffer for 45 minutes on ice, washed as already described and analyzed on a Calibur fluorescence-activated cell sorter (Becton Dickinson, North Ryde, NSW, Australia). The brightest 30% of β3-expressing cells were sorted, expanded in culture and frozen for all subsequent experiments (66cl4beta3). Fluorescence-activated cell sorting experiments were completed a minimum of two times.

### Proliferation and adhesion assays

*In vitro *proliferation assays were performed as described previously using a sulphorhodamine B colorimetric assay [40]. Proliferation of 66cl4pBabe and 66cl4beta3 cells was measured over 5 days in complete α-MEM medium with an initial cell density of 1 × 10^3^/well and five replicate wells/time point. Adhesion assays were performed in 96-well culture plates as described previously [44]. Briefly, triplicate wells were precoated overnight at 4°C with BSA (2% w/v), collagen I (20 μg/ml), collagen IV (20 μg/ml), fibronectin (10 μg/ml), or vitronectin (10 μg/ml). Osteopontin was a gift from Dr L Fisher (John Hopkins School of Medicine, MD, UAS) and was used at a concentration of 10 μg/ml. Other extracellular matrix proteins were obtained from Sigma (St-Louis, MO, USA). The cells were labeled with calcein (Molecular Probes, Eugene, OR, USA) and seeded at 2 × 10^4^/100 μl in serum-free α-MEM supplemented with 0.1% BSA, and the plates were spun at 400 × *g *for 5 minutes at 4°C. The cells were allowed to adhere for 30 minutes at 37°C. Nonadherent cells were removed by gentle washing with PBS and adherent cells were lysed with 1% Triton X-100. Where indicated, function-blocking hamster anti-mouse β3 integrin or control antibodies (10 μg/ml; Pharmingen) were used for pretreatment of the cells for 15 minutes on ice and added together with the cells to the culture wells. Adhesion was expressed as the percentage of total cell input by comparing the specific fluorescence in each well with that of 100 μl initial cell suspension in a Molecular FX fluorescence reader (Bio-Rad Laboratories, Regents Park, NSW, Australia).

For endothelial adhesion assays, bEnd.3 cells were seeded in complete DMEM medium and incubated overnight to form a confluent monolayer. Excess cells and medium were removed with PBS and calcein-labeled tumor cells (2 × 10^4^/100 μl) were added in serum-free α-MEM medium supplemented with 0.01% BSA. Culture plates were incubated for 30 minutes at 4°C and for a further 30 minutes at 37°C. Nonadherent tumor cells were removed and adhesion was measured as described earlier.

Both the proliferation and adhesion assays were completed a minimum of three times and the data are presented as the means ± standard deviation of a representative experiment performed in three (adhesion) or five (proliferation) replicate wells. The statistical differences were analyzed using the Students' *t *test; *P *< 0.01 was considered significant.

### Migration and invasion assays

Haptotactic cell migration and invasion were assayed in Transwell migration chambers (8 μm pore size; Corning, Lindfield, NSW, Australia). For haptotactic migration assays, inserts were coated on the underside with extracellular matrix molecules overnight at 4°C as described for the adhesion assay. Cells (2 × 10^5^/100 μl) were seeded into duplicate chambers and allowed to migrate for 4 hours at 37°C in the absence of serum. For invasion assays, cells were embedded in 50% Matrigel/PBS into the upper chamber and were allowed to invade and migrate toward a serum gradient (5% FCS) in the bottom well for 24 hours at 37°C. Cells were fixed in 10% buffered formalin, permeabilized in 0.1% Triton-X 100 and stained with 0.5 μg/ml 4'-6-Diamodino-2-phenylindole (Sigma). Cells remaining on the upper side of the insert were removed by wiping with cotton wool and the membrane was mounted on a glass slide. Cells that had migrated to the underside of the membrane were counted under fluorescence with 40× magnification and the average number of cells in three microscope fields/membrane was determined. Function-blocking hamster anti-mouse β3 integrin or control antibodies were used for pretreatment of the cells as described for the adhesion assay. The matrix metalloproteinase (MMP) inhibitor AG3340 (10 μM; Agouron Pharmaceuticals, San Diego, CA, USA) or vehicle alone (dimethyl sulfoxide) were used to pretreat the cells for 48 hours in standard cultures prior to the assay and were added at the same concentration to the cells in the chamber wells.

For chemotactic migration, the tibias and femurs were harvested from Balb/c mice, crushed and digested for 60 minutes with a solution of PBS, collagenase type II (6 mg/ml; Gibco, Mount Waverley, VIC, Australia) and dispase II (8 mg/ml; Roche Diagnostics, Mannheim, Germany). The cell suspension was filtered through a 70 μm nylon filter and was washed three times by centrifugation in PBS. The cell pellet was resuspended in α-MEM, 10% FCS and the cells were allowed to form a confluent monolayer in the bottom well of Transwell migration chambers. The cells were washed extensively with PBS, and then 600 μl serum-free α-MEM was added and the cells were incubated at 37°C for a further 2 hours at 37°C. Calcein-labeled tumor cells (2 × 10^5 ^in serum-free α-MEM) were pretreated or not with blocking antibodies, added to the upper well and placed above the bone cell-containing lower wells. Migration to the underside of the porous membrane was measured after four hours as described earlier.

All migration and invasion assays were completed at least three times in duplicate wells. For each duplicate, the number of migrated cells was counted in three fields of view/membrane for a total of six cell counts/condition. The results from a representative experiment are shown and expressed as the mean number of migrated cells/field ± standard deviation of six fields of view/condition. The statistical differences were analyzed using the Students' *t *test; *P *< 0.01 was considered significant.

### Animal studies

Mice were maintained in a specific pathogen-free environment with food and water freely available. All procedures involving mice accorded with National Health and Medical Research Council animal ethics guidelines. All mice used were female Balb/c (Animal Resources Centre, Perth, Australia). For intratibial injections, mice 3–4 weeks old were anesthetized by intraperitoneal injection of 40 μg ketamine/g mouse and 16 μg xylazine/g mouse. Cells (1 × 10^3^) in 20 μl PBS were injected through the proximal tibial metaphysis using a 26 G needle. Mice received the analgesic carprofen (4 μg/g mouse) at the time of injection, on the next day, and daily doses from day 10 to day 14. Mice were culled by anesthetic overdose after 14 days. For intramammary fatpad injections, mice 6–8 weeks old were anesthetized with methoxyfluorane. Cells (1 × 10^4^) in 10 μl PBS + 10 μl Matrigel were injected transdermally into the fourth (inguinal) mammary fat pad. Mice were culled by anesthetic overdose after 39 days. Primary tumors were dissected and weighed. Lungs and spines were dissected and snap frozen in liquid nitrogen. Femurs were processed for histology as described in the following.

### Analysis of metastatic tumor burden

Real-time quantitative PCR (RTQPCR) using Taqman chemistry (PE Biosystems, Foster City, CA, USA) was used to determine the relative metastatic tumor burden in mouse organs after injection of tumor cells into the fat pad [40]. Genomic DNA was extracted from organs and a multiplex reaction was performed on genomic DNA from each organ to determine the ratio of the vimentin signal (present in all cells) to the LXSN signal (retroviral LTR sequence present in stably integrated pBabe vector in tumor cells only). Primers and probes were designed using Primer Express (Applied Biosystems, Foster City, CA, USA), and were as follows (shown 5' to 3' with the corresponding Genbank accession number and the nucleotide positions of the amplicon): LXSN (GenBank accession number M28248; nucleotides 1,051–1,162 base pairs): forward, TGGCCCGACCTGAGGAA; reverse, CAGACGGAGGCGGGAACT; probe, 6FAM-CCCGTCAGGATATGTGGTTCTGGTAGGA-TAMRA; Vimentin (GenBank accession number NM_011701; nucleotides 1,146–1,226 base pairs): forward, AGCTGCTAACTACCAGGACACTATTG; reverse, CGAAGGTGACGAGCCATCTC; probe, VIC-CCTTCATGTTTTGGATCTCATCCTGCAGG-TAMRA.

Multiplex PCR reactions (1 × Taqman Universal PCR Master Mix, 50 nM vimentin forward and reverse primers, 50 nM vimentin probe, 150 nM LXSN forward and reverse primers and 50 nM LXSN probe, approximately 1 ng DNA) were cycled according to the standard protocol in an ABI PRISM 7000 Sequence Detection System (Applied Biosystems). At the end of the reaction, a fluorescence threshold was set just above baseline fluorescence levels, within the range of linear amplification. The cycle number at which fluorescence for vimentin and LXSN signals passed the threshold (*C*_T _value) was determined for each sample. The Δ*C*_T _value was determined by subtracting *C*_T(Vim) _from *C*_T(LXSN)_. Δ*C*_T _was then used to calculate the relative tumor burden (RTB) according to the equation RTB = 2^Corrρ*C*t ^× 1000, where Corrρ*C*t is the ρ *C*_t _value that includes a correction for the difference in LXSN copy number between tumor lines as determined by multiplex RTQPCR on tissue culture cells.

### Histology and tartrate-resistant acid phosphatase staining

Tissues were dissected and fixed in 10% buffered formalin, bones were decalcified in ethylenediamine tetraacetic acid and tissues were embedded in paraffin wax. Tissue sections (4 μm) were stained with H & E for morphology or were assayed for tartrate-resistant acid phosphatase activity using the Leukocyte Acid Phosphatase kit (Sigma) according to the manufacturer's instructions.

### Statistical analysis of *in vivo *data

All data for the measurement of the tumor volume and the tumor weight and for the RTQPCR analysis of relative tumor burden were derived from 15 mice/group or 10 mice/group for intratibial tumor injection. A Kolmogorov–Smirnov normality test was performed on each group to determine whether the data within groups show a normal distribution. For data that passed the normality test, the statistical differences between groups were analyzed using a Students' *t *test. Data that failed the normality test were analyzed for differences between groups using the Mann–Whitney rank sum test; *P *≤ 0.01 was considered significant.

## Results

### Expression of αvβ3 integrin in the mammary tumor lines

**Figure 1:**
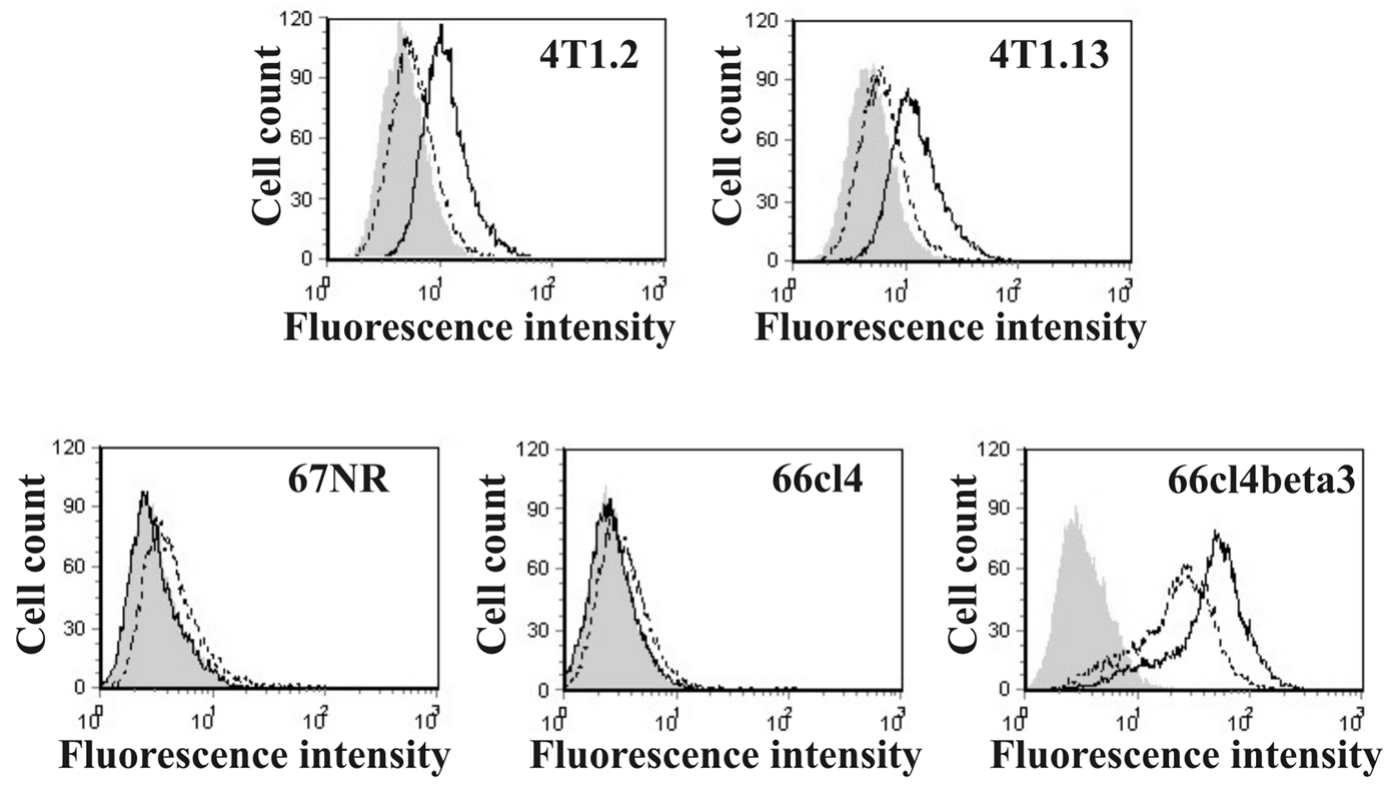
αvβ3 integrin expression in tumor lines of the metastasis model. Cell surface expression of integrin subunits was determined by measuring the specific fluorescence by flow cytometry as described in Materials and methods. Shaded area, isotype; dotted line, αv; solid line, β3.

The orthotopic breast cancer model consists of several cell lines with well-characterized metastatic profiles [40,41]. The 4T1.2 and 4T1.13 lines are highly metastatic to lymph nodes, lungs and bone following inoculation into the mammary gland. The 66cl4 line shows a low level of metastasis from the mammary fat pad to the lung, but not to bone. The 67NR line is nonmetastatic. All four lines were derived from the same spontaneous mammary carcinoma in a Balb/c/C3H mouse [42]. Expression of αvβ3 integrin in these cell lines was investigated by flow cytometry using antibodies specific to the αv and β3 subunits and an isotype-matched control antibody. Both αv and β3 subunits were expressed by 4T1.2 and 4T1.13 (Figure 1). 67NR and 66cl4 expressed low levels of the αv subunit, but not of β3 integrin. The expression of integrin αvβ3 is therefore restricted to bone metastatic lines in this model and is correlated with their aggressive metastatic phenotype.

To investigate the relationship between αvβ3 integrin expression and bone metastasis, β3 integrin expression was induced in 66cl4 cells by retroviral infection of mouse β3 cDNA. A pool of cells stably expressing high levels of β3 was selected by puromycin resistance followed by fluorescence-activated cell sorting. These cells were designated 66cl4beta3 and represent the brightest 30% of the total puromycin-resistant population. Expression of β3 integrin enhanced expression of both β3 and αv integrin subunits at the cell surface (Figure 1). The same phenomenon has been reported in MCF-7 breast cancer cells following exogenous expression of the β3 subunit [35] and may be due to enhanced transcription of the αv subunit or redistribution of a pre-existing intracellular pool. Integrin αvβ3 expression was stable in 66cl4beta3 cells for the duration of the experiments.

### αvβ3 integrin enhances spontaneous metastasis to the lung and bone

**Figure 2:**
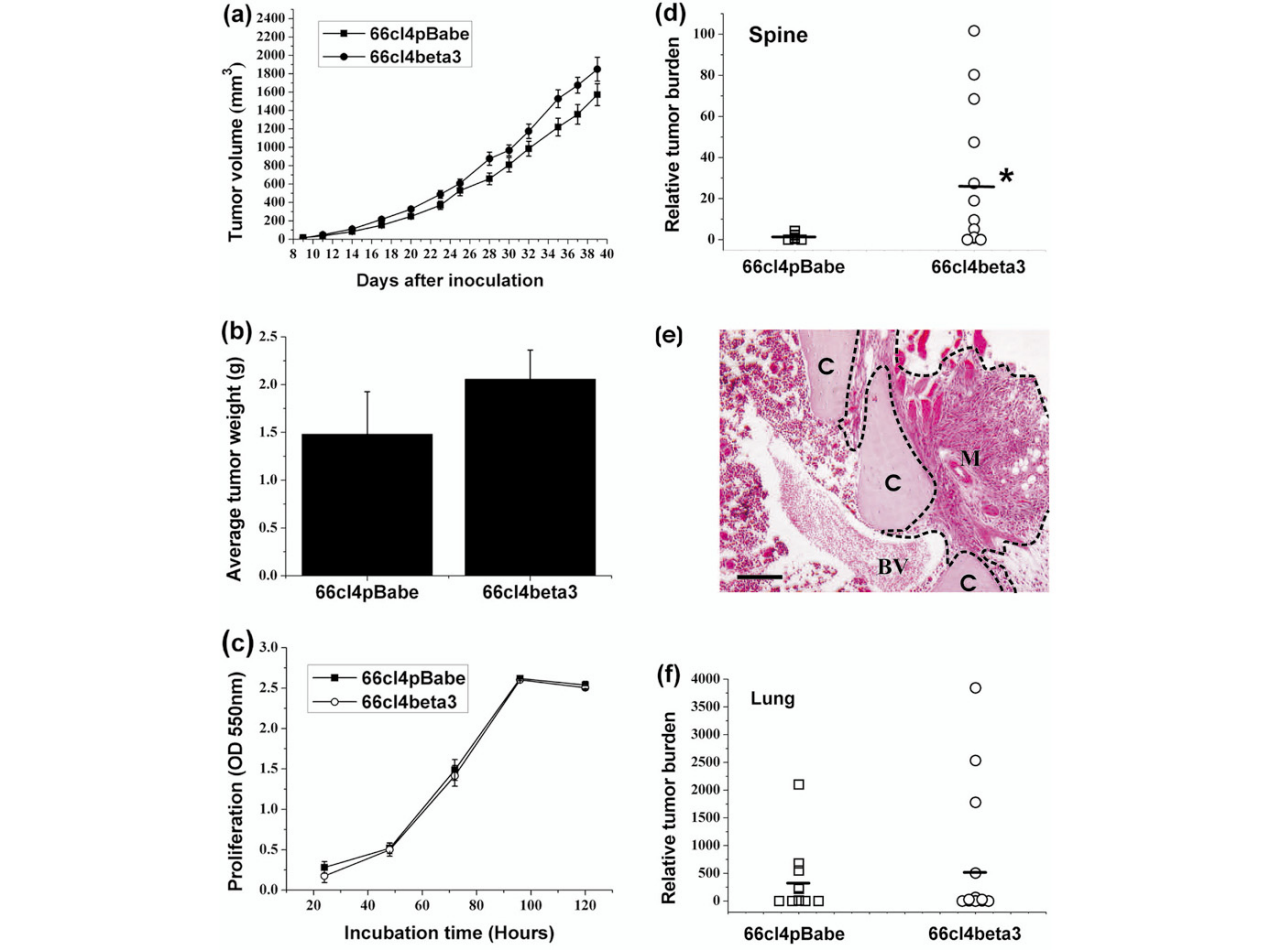
Tumor expression of αvβ3 integrin promotes spontaneous metastasis to the spine but not primary tumor growth. Cells (1 × 10^4 ^in 20 μl of 50% Matrigel) were inoculated into the mammary gland of Balb/c mice (15 mice per group). **(a) **Primary tumor volumes were measured three times per week. **(b) **Primary tumor weights were recorded on the day of harvest (day 39). **(c) ***In vitro *proliferation of 66cl4pBabe and 66cl4beta3 cells. The tumor burden in the **(d) **spine and **(f) **lungs was measured by real-time quantitative PCR as described in Materials and methods. Each point represents one mouse, but not all points are distinguishable. The average metastatic burden for each group is shown with a line. *P = 0.01. **(e) **H&E of a 66cl4beta3 spontaneous femoral metastasis. A metastatic nodule (M) in proximity of a blood vessel (BV) is seen invading through the cortical bone (C). Scale bar = 100 μm.

Given the correlation between αvβ3 expression and the ability to metastasize to bone in the metastasis model, it was of interest to determine whether expression of this integrin altered the primary tumor growth or metastasis in Balb/c mice. 66cl4pBabe or 66cl4beta3 cells were injected into the fourth mammary fat pad of Balb/c mice (*n *= 15/group). Tumors were palpable 9 days after injection. The 66cl4beta3 tumors grew slightly faster than the control group, although the difference in the growth rate (Figure 2a) or the final tumor weight at the completion of the experiment on day 39 (Figure 2b) was not statistically significant. Similarly, there was no difference between the proliferation rates of 66cl4beta3 and 66cl4pBabe cells *in vitro *(Figure 2c). The expression of αvβ3 integrin therefore does not alter tumor growth at the primary site.

Lungs and spines were harvested for analysis of the metastatic tumor burden by RTQPCR. The metastatic burden of 66cl4beta3 cells in the spine increased 20-fold over that observed with 66cl4pBabe cells, with a relative tumor burden of 25.8 ± 9.2 in 66cl4beta3 compared with 1.3 ± 0.4 in 66cl4pBabe (*P *= 0.01) (Figure 2d). Spines were chosen for quantitative RTQPCR analysis of the tumor burden in bone because the metastatic burden in the spine is typically larger and more frequent in this model than metastasis to the femur or tibia. Spontaneous metastasis of 66cl4beta3 tumors to long bones clearly occurred in some mice, however, as evidenced by histological examination of femoral sections (Figure 2e). As expected, no femoral metastases were seen in mice injected with 66cl4pBabe cells.

Similarly, there was a trend toward increased metastasis of 66cl4beta3 tumors to the lung compared with 66cl4pBabe tumors, with average relative tumor burdens of 590 ± 303 and 295 ± 163 (mean ± standard error of the mean), although the difference did not reach statistical significance (*P *= 0.32) – most probably due to the stochastic nature of spontaneous metastasis (Figure 2f). It should be noted that the high lung tumor burden in mice injected with 66cl4beta3 limited the lifespan of these mice and necessitated early termination of the experiments, therefore restricting the time available for the development of bone metastases. Attempts to extend the 39-day endpoint by resection of the primary tumors at day 14 did not increase survival time appreciably due to early onset of lung metastases.

Visual examination of all mice at harvest failed to detect visible 66cl4pBabe or 66cl4beta3 metastases in the lymph nodes, liver or kidneys (data not shown). Taken together, these results demonstrate that expression of αvβ3 integrin in breast tumor cells induces spontaneous metastasis from the mammary gland to bone in a tumor line that does not normally metastasize to this organ.

### Tumor expression of αvβ3 integrin is not required for proliferation in bone but enhances osteoclast recruitment and osteolysis

**Figure 3:**
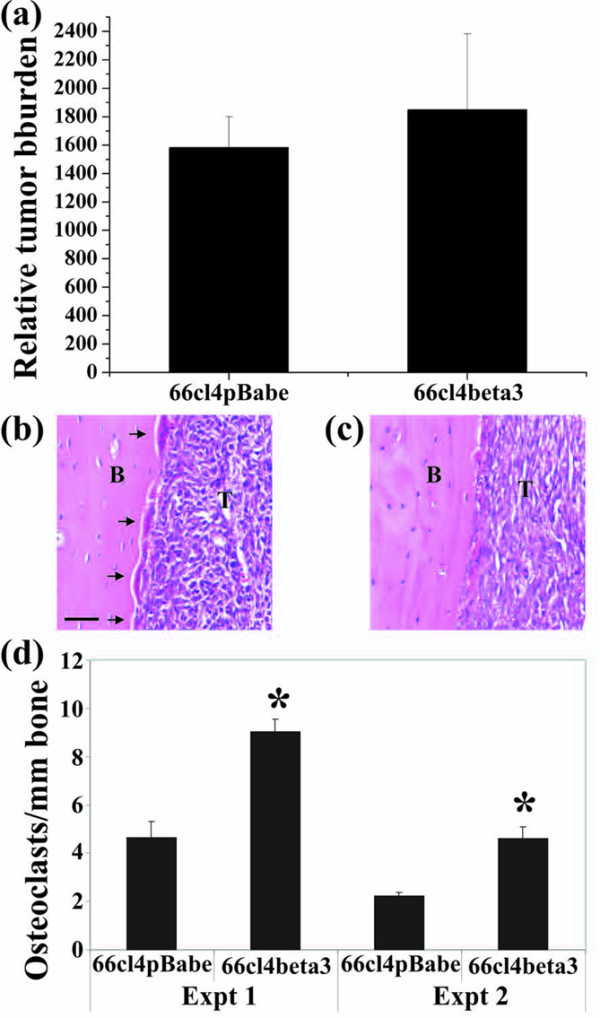
Tumor αvβ3 integrin enhances osteoclast recruitment and osteolysis following intratibial injection of tumor cells. **(a) **The relative tumor burden was measured by real-time quantitative PCR 14 days following direct intratibial injection of 66cl4pBabe and 66cl4beta3 cells. Representative H&E-stained sections through tibias injected with **(b) **66cl4beta3 or **(c) **66cl4pBabe cells. Osteoclasts are marked with arrows. B, bone; T, tumor. Scale bar = 50 μm. **(d) **Serial sections adjacent to those stained by H&E were stained for tartrate-resistant acid phosphatase (TRAP) activity, and the active osteoclasts (TRAP-positive) present per millimeter of bone in tibias injected with either 66cl4beta3 or 66cl4pBabe cells were counted. Results of two independent experiments are shown. *n *= 4 mice/group per experiment *P < 0.001.

To determine whether expression of αvβ3 integrin is required for proliferation in bone, 66cl4beta3 or 66cl4pBabe cells were injected directly into the tibia. Mice were culled after 14 days and the tibias were processed for quantitative measurement of the tumor burden by RTQPCR or for histological examination of serial sections taken from three levels through each tibia, and were stained with H&E to confirm tumor growth and to measure the length of the bone surface adjacent to the tumor nodule. As shown in Figure 3a–c, the extent of the tumor deposit was similar for 66cl4pBabe and 66cl4beta3 cells, indicating that tumor expression of αvβ3 integrin does not directly confer a proliferative advantage in bone and that the failure of 66cl4pBabe to metastasize spontaneously to bone is not due to its inability to grow in this organ.

Osteoclast-mediated resorption of bone is intimately linked to growth of breast cancer in bone [45]. Recruitment and activation of osteoclasts in proximity of the metastatic nodule is well documented and thought to contribute indirectly to metastatic growth in bone via the release of mitogenic factors from the bone matrix [46]. To determine whether expression of αvβ3 alters tumor-associated bone resorption, the ability of 66cl4beta3 cells to influence osteoclastogenesis within the bone microenvironment following intratibial injection of the cells was investigated. Sections adjacent to those stained with H&E were stained for tartrate-resistant acid phosphatase activity to detect active osteoclasts (Figure 3a,b). The density of osteoclasts (osteoclasts/mm) was determined at each level through each tibia. Analysis of tibias injected with 66cl4beta3 and 66cl4pBabe cells in two independent experiments showed that osteoclast density was approximately twofold higher in the 66cl4beta3 tibial tumors (Figure 3c). Osteoclasts were present at the interface between the bone and tumor cells and also at the interface between the tumor and the bone marrow. Cortical bone surfaces adjacent to 66cl4beta3 tumor nodules showed extensive resorption (Figure 3a). In contrast, fewer osteoclasts and negligible bone resorption were observed in the proximity of the 66cl4pBabe tumor deposits (Figure 3b).

### Expression of αvβ3 integrin stimulates tumor cell adhesion to vitronectin

**Figure 4:**
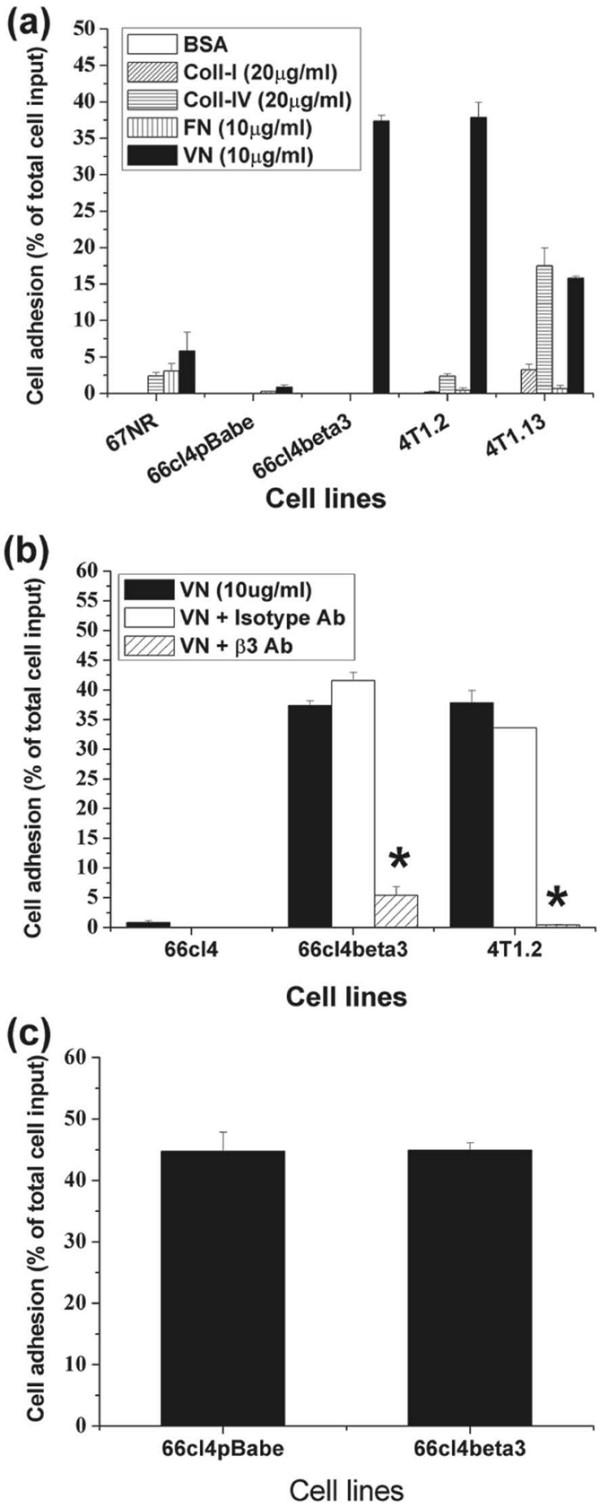
αvβ3 integrin mediates mammary tumor cell adhesion to vitronectin but not to endothelial cells. **(a) **Short-term adhesion to different matrices. **(b) **Effect of neutralizing antibodies against β3 integrin on short-term adhesion to vitronectin. **(c) **Adhesion of 66cl4pBabe and 66cl4beta3 cells to a monolayer of bEnd.3 endothelial cells in the absence of serum. The experiments were repeated at least twice and the results represent the mean ± standard deviation of a representative experiment performed in triplicate wells. *P < 0.001.

To explore the mechanisms by which αvβ3 integrin enhanced metastasis to bone, the ability of the cells to adhere to extracellular matrix molecules was investigated (Figure 4). In this short-term adhesion assay (30 minutes), none of the lines adhered to the control BSA coating. The 67NR line adhered only weakly to vitronectin, fibronectin and collagen IV (Figure 4a). None of the substrates tested promoted rapid adhesion of the weakly metastatic line 66cl4pBabe. Exogenous expression of αvβ3 integrin in 66cl4 cells, however, resulted in markedly increased adhesion to vitronectin, but not to other matrices (Figure 4a). Bone metastatic 4T1.2 cells similarly adhered most avidly to vitronectin but poorly to other substrates, whereas 4T1.13 cells showed moderate binding to collagen IV and vitronectin (Figure 4a). In adhesion assays extended over several hours, the cells adhered to all these matrices (data not shown). Treatment of 66cl4beta3 and 4T1.2 cells with a function-blocking anti-β3 integrin antibody, but not with an isotype-matched control antibody, substantially inhibited their adhesion to vitronectin, indicating that adhesion to vitronectin was mediated specifically via αvβ3 integrin (Figure 4b). A similar inhibition of binding to vitronectin was observed in the 4T1.13 line (data not shown).

Additional experiments were conducted to assess whether tumor αvβ3 may contribute to attachment to endothelial cells, a process required for intravasation/extravasation of metastatic tumor cells *in vivo*. We compared 66cl4pBabe and 66cl4beta3 cells for their ability to attach to a confluent monolayer of microvascular endothelial bEnd.3 cells in the absence of serum. As shown in Figure 4c, both 66cl4pBabe and 66cl4beta3 cells adhered to bEnd.3 cells to the same extent, indicating that αvβ3 is not required for their adhesion to endothelial cells.

### αvβ3 integrin promotes migration and invasion of 66cl4 cells

**Figure 5:**
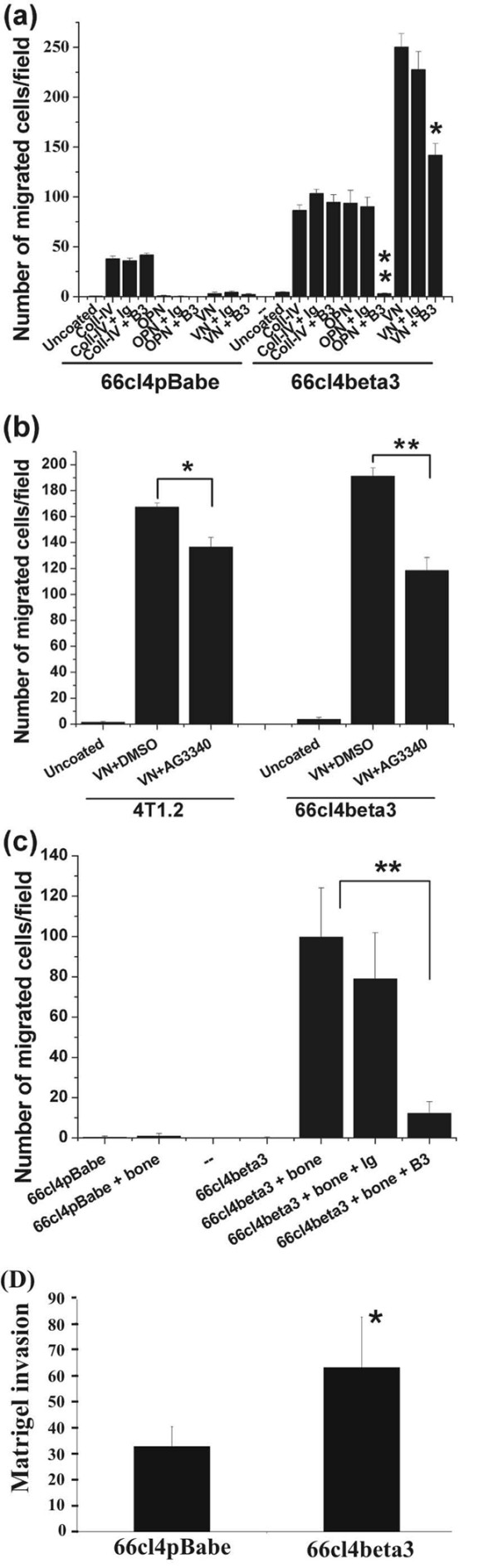
Expression of αvβ3 integrin promotes tumor cell migration and invasion. Migration and Matrigel invasion assays were performed in Transwell migration chambers (8 μm pore size). **(a) **Haptotactic migration in response to collagen IV, osteopontin and vitronectin and the effect of neutralizing β3 antibodies. **(b) **Effect of the matrix metalloproteinase inhibitor AG3340 (10 μM) on the haptotactic migration of 4T1.2 and 66cl4beta3 cells. **(c) **Chemotactic migration of 66cl4pBabe and 66cl4beta3 cells towards a monolayer of bone stromal cells seeded in the lower chamber. **(d) **Invasion of cells through Matrigel in response to a serum chemotactic gradient. All assays were performed in duplicate wells and the cells counted from three fields of view/membrane at 40× magnification (a, b and d) or 20× magnification (c). The experiments were repeated at least twice and the data represent the mean number of migrated cells ± standard deviation of six fields of view/condition from a representative experiment performed in duplicate wells. *P < 0.01, **P < 0.001.

To metastasize successfully, cells need to acquire a motile phenotype and to invade through the basement membrane and surrounding stroma. Site-specific metastasis has been proposed to be regulated, in part, by adhesion to and migration towards the ECM expressed at metastatic sites. To explore the role of αvβ3 integrin in these processes, we first compared the haptotactic migration of 66cl4pBabe and 66cl4beta3 cells towards specific extracellular matrix substrates coated on the underside of the porous membranes of the transwell inserts (Figure 5a). The inserts were placed in wells containing serum-free medium. Collagen IV stimulated the haptotactic migration of 66cl4pBabe cells with a further enhancement in 66cl4beta3 cells. This migration, however, was not blocked by antibodies targeting β3 integrin (Figure 5a). Control 66cl4pBabe cells showed no haptotactic migration towards osteopontin, an abundant αvβ3 integrin ligand in bone, presumably due to their lack of αvβ3 expression. Consistent with this, 66cl4beta3 cells were strongly migratory towards osteopontin in a process blocked completely by neutralizing antibodies against integrin β3. Migration towards vitronectin was also strongly stimulated by β3 integrin expression in 66cl4beta3 cells and was partially inhibited by neutralization of αvβ3 (Figure 5a).

The cooperative role of integrins and MMPs in tumor cell migration and invasion has been extensively documented. We have previously reported that all of the tumor lines of the model express similar levels of MT1-MMP but none express detectable MMP-2. The bone metastatic 4T1.2 cells express significantly higher levels of MMP-9 than 66cl4 or 67NR cells [47]. We confirmed here by gelatin zymography that exogenous expression of αvβ3 integrin in 66cl4 cells did not induce MMP-2 or alter the profile of MMP-9 expression when the cells were plated on plastic or on vitronectin (data not shown), suggesting that increased haptotactic migration of 66cl4beta3 cells towards vitronectin occurred independent of changes in MMP-2 and MMP-9 expression levels.

To determine whether protease activity is required for vitronectin-mediated haptotactic migration of 4T1.2 and 66cl4beta3 cells, we tested the effect of the broad-spectrum hydroxamate MMP inhibitor Prinomastat (AG3340) in the haptotaxis assay. For these experiments, the cells were pretreated with low doses of AG3340 (10 μM) for 48 hours prior to seeding into uncoated or vitronectin-coated wells. At this concentration, no toxicity was observed in vehicle (dimethyl sulfoxide)-treated cells or AG3340-treated cells. As seen earlier, neither 4T1.2 nor 66cl4beta3 migrated in the absence of coated substrate but their migration was strongly induced by the presence of vitronectin (Figure 5b). Importantly, pretreatment with AG3340 inhibited 4T1.2 and 66cl4beta3 cell migration by 23% and 40% respectively, compared with control dimethyl sulfoxide alone. αvβ3 integrin-mediated migration of bone metastatic mammary carcinoma cells towards vitronectin is therefore dependent, in part, on extracellular protease activity.

To determine whether secreted factors produced in the bone microenvironment may also contribute to homing of 66cl4beta3 cells to bone, we compared the effect of bone stromal cells isolated from tibias and femurs of Balb/c mice on the chemotactic migration of 66cl4pBabe and 66cl4beta3 cells in the absence of serum. As shown in Figure 5c, neither line migrated in the absence of bone cells in the bottom chamber. In contrast, while chemotactic migration of 66cl4pBabe was negligible when stromal cells were added to the lower chamber, 66cl4beta3 efficiently migrated under these conditions. Importantly, chemotactic migration of 66cl4beta3 cells was almost completely abrogated by treatment with β3-neutralizing antibodies but not by control antibodies (Figure 5c). The invasive capacity of 66cl4pBabe and 66cl4beta3 cells was assessed by their ability to move through a Matrigel barrier in response to a chemotactic gradient provided by FCS in the lower chamber. The αvβ3 integrin-expressing 66cl4beta3 cells showed enhanced invasion through Matrigel compared with 66cl4pBabe (Figure 5d).

Taken together, these results demonstrate that expression of αvβ3 integrin promotes mammary carcinoma cell adhesion to and haptotactic migration towards bone matrix proteins, enhances their responsiveness to bone-derived soluble factor and increases their invasive properties. Migration towards vitronectin appears to be partially dependent on MMP activity.

## Discussion

Numerous *in vitro *and *in vivo *studies have documented the correlation between tumor expression of αvβ3 integrin and the bone metastatic potential of breast tumors and other human tumors [7,25,26,48-50]. Our observation that only aggressive and bone metastatic mammary carcinoma cell lines of our murine model express this receptor is consistent with these studies. The precise role of tumor-specific αvβ3 integrin and its relative contribution to primary tumor growth and bone metastasis remains unclear. This is due in part to the lack of clinically relevant syngeneic models of spontaneous breast cancer metastasis that recapitulate the entire metastatic cascade from the mammary gland to bone. We report in the present article that exogenous expression of αvβ3 integrin in mammary carcinoma cells that do not normally metastasize to bone is sufficient to promote their spontaneous metastasis to this organ. To our knowledge, these data provide the first direct *in vivo *evidence that tumor αvβ3 integrin contributes to the spontaneous metastasis of breast tumors from the mammary gland to bone.

Increased bone metastasis occurred without apparent effect on 66cl4beta3 primary tumor growth *in vivo *or proliferation *in vitro*. These observations contrast with some [11,20], but not all [37], studies in xenograft models showing inhibition of mammary tumor growth by αvβ3 antagonists. Reduced angiogenesis observed in these tumors suggests that αvβ3 inhibitors impair tumor growth primarily by targeting the tumor neovasculature rather than the tumor cells. Unlike these studies, our approach addressed specifically the role of tumor αvβ3. Comparable growth of 66cl4pBabe and 66cl4beta3 tumors indicates that tumor αvβ3 is unlikely to promote spontaneous bone metastasis through stimulation of tumor cell proliferation at the primary site. Instead, results from *in vitro *assays suggest that tumor αvβ3 may have multiple roles downstream of primary tumor growth that contribute to enhanced metastasis to bone. This conclusion is supported by the observation that the selective αvβ3 antagonist S247 inhibits the metastasis of αvβ3-expressing MDA-MB-435 to bone in the experimental metastasis model that bypasses the formation of a primary tumor [39].

While our results show increased spontaneous metastasis predominantly to bone and are consistent with data from experimental models, they do not preclude that tumor αvβ3 may enhance metastasis to several organs in part through its effect on earlier stages of metastasis, such as escape from the primary tumor and intravasation. For instance, we found that 66cl4beta3 cell migration towards collagen IV and invasion through Matrigel were substantially increased compared with that of 66cl4pBabe cells (see Figure 5a). Both these responses are likely to be important in early dissemination of metastatic cells from the mammary gland and may explain also the trend towards increased spontaneous metastasis to lung observed in our model (Figure 2e). This would be consistent also with the inhibitory effect of S247 reported on the spontaneous metastasis of MDA-MB-435 cells to the lung despite its lack of effect on primary tumor growth in the mammary gland [37].

The mechanisms leading to increased collagen-IV-mediated migration of 66cl4beta3 cells are unclear but could involve MT1-MMP-dependent regulation of cross-talk between αvβ3 integrin and the α2β1 collagen receptor, as reported previously in human breast carcinoma cells [51]. In particular, these authors noted that MCF-7 cells expressing both MT1-MMP and αvβ3 integrin were more migratory towards collagen I than MCF-7 cells expressing only MT1-MMP. By analogy, 66cl4beta3 cells express both αvβ3 and MT1-MMP whereas 66cl4pBabe express only MT1-MMP ([47] and this study).

In agreement with previous reports [25-27,30,38], we found that expression of αvβ3 integrin in 66cl4beta3 cells dramatically enhanced their αvβ3-dependent adhesion and haptotactic migration towards bone matrix proteins. It is probable that these interactions are critical for homing of breast tumor cells to bone. Migration towards vitronectin was partially inhibited by the MMP inhibitor AG3340, suggesting cooperation between αvβ3 and MMPs to promote haptotactic migration. Functional cooperation between αvβ3 integrin and several MMPs, including MMP-2, MMP-9 and MT1-MMP, has been demonstrated previously [5,29,31,52]. 66cl4 cells express similar levels of MT1-MMP but significantly less MMP-9 than the bone metastatic 4T1.2 cells [47]. Neither line expresses detectable levels of MMP-2. Since we did not detect any significant changes in the levels of MMP-2 or MMP-9 activity between 66cl4beta3 and 66cl4pBabe cells by gelatin zymography, cooperation between αvβ3 and MMP-2/9 is unlikely to account for the enhanced haptotactic response observed in 66cl4beta3 cells.

Interestingly, Deryugina and colleagues reported that expression of αvβ3 in MCF-7 cells is insufficient alone to promote their migration towards vitronectin and requires the proteolytic processing of the αvβ3 receptor by MT1-MMP to migrate [29,53]. Short-term treatment of MCF-7 cells coexpressing MT1-MMP and αvβ3 with AG3340 blocked direct proteolysis of vitronectin by MT1-MMP and enhanced migration. In contrast, due to the slow turnover of αvβ3, long-term treatment was required to replace existing pools of αvβ3 proteolytically activated by MT1-MMP at the cell surface and to inhibit migration. Similarly, long-term treatment (48 hours) with AG3340 was required to block 4T1.2 and 66cl4beta3 cell migration (see Figure 5b). Although we cannot completely rule out a potential interaction between αvβ3 and MMP-9 in 66cl4beta3 cells, these observations suggest that functional activation of αvβ3 by MT1-MMP may be more relevant to the haptotactic response of 66cl4beta3 cells to vitronectin.

While our results clearly implicate tumor αvβ3 in breast cancer metastasis to bone, it should be noted that the metastatic burden observed with 66cl4beta3 was not as high as that typically seen using the more aggressive 4T1.2 and 4T1.3 lines [40]. There therefore does not appear to be a direct correlation between the level of αvβ3 expression and the extent of metastasis to bone. Presumably, the level of αvβ3 integrin found in 4T1.2/4T1.13 cells is sufficient to promote metastasis to bone, and expression above this level (as seen in 66cl4beta3) does not offer additional benefits. Moreover, unlike 4T1.2/4T1.13 lines, we failed to detect any spontaneous 66cl4beta3 metastases in the lymph nodes, kidney or liver. A probable explanation for these differences is that other factors not present in 66cl4beta3 cells cooperate with αvβ3 integrin and contribute to the high metastatic phenotype of 4T1.2/4T1.13 lines. Although we found no evidence implicating MMP-9 in the 66cl4beta3 migratory response to vitronectin, it is tempting to speculate that enhancing the level of MMP-9 expression (as seen in 4T1.2 and 4T1.13 cells) together with expression of αvβ3 in 66cl4beta3 cells may be sufficient to achieve the high metastatic burden observed with 4T1.2 and 4T1.13 tumors. Alternatively, coexpression of αvβ3 and its ECM ligand may be required to further enhance the 66cl4beta3 metastatic potential as demonstrated in 21NT human xenografts [34]. Consistent with this, cDNA microarray analysis of primary tumors of our model revealed that several ECM-related genes are more highly expressed in bone metastatic lines (4T1.2, 4T1.13) compared with weakly metastatic (66cl4) and nonmetastatic (67NR) lines [40]. The cooperative role of some of these ECMs and αvβ3 integrin is under investigation.

Tumor αvβ3 has been proposed to enhance metastasis by facilitating tumor cell arrest in the vasculature through interaction with platelets and adhesion to the subendothelial matrix [14,32]. 66cl4pBabe and 66cl4beta3 cells adhered equally well to endothelial cells, indicating that receptors other than αvβ3 integrin mediate their attachment to the endothelium under our assay conditions. It should be noted, however, that the assays were conducted in the absence of serum and thus a role for αvβ3-mediated tumor–platelet interaction under blood flow conditions cannot be ruled out. Further work will be required to address this possibility.

Exogenous expression of αvβ3 integrin dramatically enhanced the chemotactic migration of 66cl4beta3 cells towards soluble factors secreted by bone stromal cells. Almost complete inhibition of chemotaxis by β3-blocking antibodies indicates that the antibody either prevents binding of αvβ3 receptor to a soluble ECM ligand and/or interferes with the association of αvβ3 and a chemotactic receptor. Several factors produced in the bone stromal microenvironment have been shown to promote chemotactic migration, including insulin-like growth factors, platelet-derived growth factor (PDGF) and stromal cell derived factor 1 (sdf-1/CXCL12) [45,54-56]. While we have yet to determine the specific bone stromal factor stimulating the 66cl4beta3 cell chemotactic response, PDGF may be of particular interest as αvβ3 integrin has been shown to associate with the PDGF receptor β and to enhance chemotactic migration in response to PDGF stimulation [57-59]. Moreover, PDGF is a potent mitogen for breast tumor cells and blocking PDGF receptor signaling in these cells inhibits their growth in bone and associated osteolysis [60].

The observations that 66cl4pBabe tumors were able to grow when injected directly into the tibia clearly demonstrate that tumor αvβ3 is not required for proliferation of breast tumor cells in bone. Moreover, the fact that they proliferated to the same extent as 66cl4beta3 tumors despite evidence of osteoclast recruitment or large lytic lesions indicates that extensive bone degradation is not required for metastatic growth to occur in bone. The increased recruitment of osteoclasts in the proximity of 66cl4beta3 tumor nodules and evidence of osteolysis at the tumor–bone interface, however, are consistent with the vicious cycle theory, and suggesting that tumor αvβ3 may play an indirect role in promoting the growth of breast cancer cells that spontaneously metastasize to bone through enhanced osteoclast-mediated bone resorption and release of mitogenic factors from the bone matrix [46]. Engagement of αvβ3 integrin with bone ECM proteins may induce production of an osteoclast-stimulating factor, such as RANKL or colony stimulating factor 1, by tumor cells or by bone marrow cells. Alternatively, tumor αvβ3 may promote osteoclastogenesis through interaction with activated platelets in the bone microenvironment [61]. While Boucharaba and colleagues did not specifically investigate the role of tumor αvβ3, they showed that interaction of human MDA-MB-231/B02 breast tumor cells with platelets promotes osteoclast-mediated bone resorption through the release of cytokines (IL-6 and IL-8) in response to platelet-derived lysophosphatidic acid. Further work will be required to elucidate the mechanism by which tumor αvβ3 enhances osteoclastogenesis. Assays that measure the production of such factors in cocultures of breast cancer cells and bone marrow stromal cells may help to elucidate the mechanism by which tumor αvβ3 integrin promotes the formation of osteolytic lesions.

## Conclusion

We have shown for the first time that tumor αvβ3 integrin contributes to the spontaneous metastasis of breast tumors to bone. Results from our investigation highlight the critical role of this integrin in homing of breast tumor cells to bone through enhanced chemotactic migration and haptotactic migration as well as in recruiting activated osteoclasts at metastatic lesions. The addition of an inhibitor specifically targeting tumor αvβ3 to existing treatments may provide greater therapeutic benefits for the treatment of patients with advanced breast cancer.

## Abbreviations

BSA = bovine serum albumin; DMEM = Dulbecco's modified Eagle's medium; ECM = extracellular matrix; FCS = fetal calf serum; H&E = haematoxylin & eosin; α-MEM = alpha minimal essential medium; MMP = matrix metalloproteinase; PBS = phosphate-buffered saline; PCR = polymerase chain reaction; PDGF = platelet-derived growth factor; RTB = relative tumor burden; RTQPCR = real-time quantitative polymerase chain reaction.

## Competing interests

The authors declare that they have no competing interests.

## Authors' contributions

The authors' contributions to this research work are reflected in the order shown, with the exception of EKS and NP who contributed equally to the majority of the *in vitro *and *in vivo *experimental work and preparation of the manuscript. KLS assisted with the monitoring of the mice, measurement of tumors and processing of tissues for real-time PCR. JC performed the endothelial adhesion assay. DKH performed the histological analysis. JMM contributed to the experimental design of the project. RLA conceived the study and participated in its design and coordination. All authors have read and approved the final manuscript.

## References

[B1] Coleman RE, Smith P, Rubens RD (1998). Clinical course and prognostic factors following bone recurrence from breast cancer. Br J Cancer.

[B2] Coleman RE, Rubens RD (1987). The clinical course of bone metastases from breast cancer. Br J Cancer.

[B3] Giancotti FG, Ruoslahti E (1999). Integrin signaling. Science.

[B4] Hewitt RE, Powe DG, Morrell K, Balley E, Leach IH, Ellis IO, Turner DR (1997). Laminin and collagen IV subunit distribution in normal and neoplastic tissues of colorectum and breast. Br J Cancer.

[B5] Rolli M, Fransvea E, Pilch J, Saven A, Felding-Habermann B (2003). Activated integrin alphavbeta3 cooperates with metalloproteinase MMP-9 in regulating migration of metastatic breast cancer cells. Proc Natl Acad Sci USA.

[B6] Zutter MM, Sun H, Santoro SA (1998). Altered integrin expression and the malignant phenotype: the contribution of multiple integrated integrin receptors. J Mammary Gland Biol Neoplasia.

[B7] Liapis H, Flath A, Kitazawa S (1996). Integrin alpha V beta 3 expression by bone-residing breast cancer metastases. Diagn Mol Pathol.

[B8] Cooper CR, Chay CH, Pienta KJ (2002). The role of alpha(v)beta(3) in prostate cancer progression. Neoplasia.

[B9] Eliceiri BP, Cheresh DA (2001). Adhesion events in angiogenesis. Curr Opin Cell Biol.

[B10] Brooks PC, Clark RA, Cheresh DA (1994). Requirement of vascular integrin alpha v beta 3 for angiogenesis. Science.

[B11] Brooks PC, Stromblad S, Klemke R, Visscher D, Sarkar FH, Cheresh DA (1995). Antiintegrin alpha v beta 3 blocks human breast cancer growth and angiogenesis in human skin. J Clin Invest.

[B12] Varner JA, Nakada MT, Jordan RE, Coller BS (1999). Inhibition of angiogenesis and tumor growth by murine 7E3, the parent antibody of c7E3 Fab (abciximab; ReoPro). Angiogenesis.

[B13] Taverna D, Moher H, Crowley D, Borsig L, Varki A, Hynes RO (2004). Increased primary tumor growth in mice null for beta3- or beta3/beta5-integrins or selectins. Proc Natl Acad Sci USA.

[B14] Felding-Habermann B, O'Toole TE, Smith JW, Fransvea E, Ruggeri ZM, Ginsberg MH, Hughes PE, Pampori N, Shattil SJ, Saven A, Mueller BM (2001). Integrin activation controls metastasis in human breast cancer. Proc Natl Acad Sci USA.

[B15] Bakewell SJ, Nestor P, Prasad S, Tomasson MH, Dowland N, Mehrotra M, Scarborough R, Kanter J, Abe K, Phillips D, Weilbaecher KN (2003). Platelet and osteoclast beta3 integrins are critical for bone metastasis. Proc Natl Acad Sci USA.

[B16] Max R, Gerritsen RR, Nooijen PT, Goodman SL, Sutter A, Keilholz U, Ruiter DJ, De Waal RM (1997). Immunohistochemical analysis of integrin alpha vbeta3 expression on tumor-associated vessels of human carcinomas. Int J Cancer.

[B17] Gasparini G, Brooks PC, Biganzoli E, Vermeulen PB, Bonoldi E, Dirix LY, Ranieri G, Miceli R, Cheresh DA (1998). Vascular integrin alpha(v)beta3: a new prognostic indicator in breast cancer. Clin Cancer Res.

[B18] Nemeth JA, Cher ML, Zhou Z, Mullins C, Bhagat S, Trikha M (2003). Inhibition of alpha(v)beta3 integrin reduces angiogenesis, bone turnover, and tumor cell proliferation in experimental prostate cancer bone metastases. Clin Exp Metastasis.

[B19] Silletti S, Kessler T, Goldberg J, Boger DL, Cheresh DA (2001). Disruption of matrix metalloproteinase 2 binding to integrin alpha vbeta 3 by an organic molecule inhibits angiogenesis and tumor growth *in vivo*. Proc Natl Acad Sci USA.

[B20] Zhou Q, Sherwin RP, Parrish C, Richters V, Groshen SG, Tsao-Wei D, Markland FS (2000). Contortrostatin, a dimeric disintegrin from Agkistrodon contortrix contortrix, inhibits breast cancer progression. Breast Cancer Res Treat.

[B21] Reynolds LE, Wyder L, Lively JC, Taverna D, Robinson SD, Huang X, Sheppard D, Hynes RO, Hodivala-Dilke KM (2002). Enhanced pathological angiogenesis in mice lacking beta3 integrin or beta3 and beta5 integrins. Nat Med.

[B22] Tucker GC (2002). Inhibitors of integrins. Curr Opin Pharmacol.

[B23] Pignatelli M, Cardillo MR, Hanby A, Stamp GW (1992). Integrins and their accessory adhesion molecules in mammary carcinomas: loss of polarization in poorly differentiated tumors. Hum Pathol.

[B24] Clezardin P, Frappart L, Clerget M, Pechoux C, Delmas PD (1993). Expression of thrombospondin (TSP1) and its receptors (CD36 and CD51) in normal, hyperplastic, and neoplastic human breast. Cancer Res.

[B25] Wong NC, Mueller BM, Barbas CF, Ruminski P, Quaranta V, Lin EC, Smith JW (1998). Alphav integrins mediate adhesion and migration of breast carcinoma cell lines. Clin Exp Metastasis.

[B26] van der P, Vloedgraven H, Papapoulos S, Lowick C, Grzesik W, Kerr J, Robey PG (1997). Attachment characteristics and involvement of integrins in adhesion of breast cancer cell lines to extracellular bone matrix components. Lab Invest.

[B27] Sung V, Stubbs JT, Fisher L, Aaron AD, Thompson EW (1998). Bone sialoprotein supports breast cancer cell adhesion proliferation and migration through differential usage of the alpha(v)beta3 and alpha(v)beta5 integrins. J Cell Physiol.

[B28] Noti JD (2000). Adherence to osteopontin via alphavbeta3 suppresses phorbol ester-mediated apoptosis in MCF-7 breast cancer cells that overexpress protein kinase C-alpha. Int J Oncol.

[B29] Deryugina EI, Bourdon MA, Jungwirth K, Smith JW, Strongin AY (2000). Functional activation of integrin alpha V beta 3 in tumor cells expressing membrane-type 1 matrix metalloproteinase. Int J Cancer.

[B30] Bartsch JE, Staren ED, Appert HE (2003). Adhesion and migration of extracellular matrix-stimulated breast cancer. J Surg Res.

[B31] Deryugina EI, Ratnikov B, Monosov E, Postnova TI, DiScipio R, Smith JW, Strongin AY (2001). MT1-MMP initiates activation of pro-MMP-2 and integrin alphavbeta3 promotes maturation of MMP-2 in breast carcinoma cells. Exp Cell Res.

[B32] Gomes N, Vassy J, Lebos C, Arbeille B, Legrand C, Fauvel-Lafeve F (2004). Breast adenocarcinoma cell adhesion to the vascular subendothelium in whole blood and under flow conditions: effects of alphavbeta3 and alphaIIbbeta3 antagonists. Clin Exp Metastasis.

[B33] Kanamori M, Vanden Berg SR, Bergers G, Berger MS, Pieper RO (2004). Integrin beta3 overexpression suppresses tumor growth in a human model of gliomagenesis: implications for the role of beta3 overexpression in glioblastoma multiforme. Cancer Res.

[B34] Furger KA, Allan AL, Wilson SM, Hota C, Vantyghem SA, Postenka CO, Al-Katib W, Chambers AF, Tuck AB (2003). Beta(3) integrin expression increases breast carcinoma cell responsiveness to the malignancy-enhancing effects of osteopontin. Mol Cancer Res.

[B35] Pereira JJ, Meyer T, Docherty SE, Reid HH, Marshall J, Thompson EW, Rossjohn J, Price JT (2004). Bimolecular interaction of insulin-like growth factor (IGF) binding protein-2 with alphavbeta3 negatively modulates IGF-I-mediated migration and tumor growth. Cancer Res.

[B36] Felding-Habermann B, Fransvea E, O'Toole TE, Manzuk L, Faha B, Hensler M (2002). Involvement of tumor cell integrin alpha v beta 3 in hematogenous metastasis of human melanoma cells. Clin Exp Metastasis.

[B37] Shannon KE, Keene JL, Settle SL, Duffin TD, Nickols MA, Westlin M, Schroeter S, Ruminski PG, Griggs DW (2004). Anti-metastatic properties of RGD-peptidomimetic agents S137 and S247. Clin Exp Metastasis.

[B38] Pecheur I, Peyruchaud O, Serre CM, Guglielmi J, Voland C, Bourre F, Margue C, Cohen-Solal M, Buffet A, Kieffer N (2002). Integrin alpha(v)beta3 expression confers on tumor cells a greater propensity to metastasize to bone. Faseb J.

[B39] Harms JF, Welch DR, Samant RS, Shevde LA, Miele ME, Babu GR, Goldberg SF, Gilman VR, Sosnowski DM, Campo DA (2004). A small molecule antagonist of the alpha(v)beta3 integrin suppresses MDA-MB-435 skeletal metastasis. Clin Exp Metastasis.

[B40] Eckhardt BL, Parker BS, van Laar RK, Restall CM, Natoli AL, Tavaria MD, Stanley KL, Sloan EK, Moseley JM, Anderson RL (2005). Genomic analysis of a spontaneous model of breast cancer metastasis to bone reveals a role for the extracellular matrix. Mol Cancer Res.

[B41] Lelekakis M, Moseley JM, Martin TJ, Hards D, Williams E, Ho P, Lowen D, Javni J, Miller FR, Slavin J (1999). A novel orthotopic model of breast cancer metastasis to bone. Clin Exp Metastasis.

[B42] Aslakson CJ, Miller FR (1992). Selective events in the metastatic process defined by analysis of the sequential dissemination of subpopulations of a mouse mammary tumor. Cancer Res.

[B43] Morgenstern JP, Land H (1990). A series of mammalian expression vectors and characterisation of their expression of a reporter gene in stably and transiently transfected cells. Nucleic Acids Res.

[B44] Pouliot N, Saunders NA, Kaur P (2002). Laminin 10/11: an alternative adhesive ligand for epidermal keratinocytes with a functional role in promoting proliferation and migration. Exp Dermatol.

[B45] Sloan EK, Anderson RL (2002). Genes involved in breast cancer metastasis to bone. Cell Mol Life Sci.

[B46] Kakonen SM, Mundy GR (2003). Mechanisms of osteolytic bone metastases in breast carcinoma. Cancer.

[B47] Tester AM, Ruangpanit N, Anderson RL, Thompson EW (2000). MMP-9 secretion and MMP-2 activation distinguish invasive and metastatic sublines of a mouse mammary carcinoma system showing epithelial-mesenchymal transition traits. Clin Exp Metastasis.

[B48] Takayama S, Ishii S, Ikeda T, Masamura S, Doi M, Kitajima M (2005). The relationship between bone metastasis from human breast cancer and integrin alpha(v)beta3 expression. Anticancer Res.

[B49] Kitazawa S, Maeda S (1995). Development of skeletal metastases. Clin Orthop Relat Res.

[B50] Putz E, Witter K, Offner S, Stosiek P, Zippelius A, Johnson J, Zahn R, Riethmuller G, Pantel K (1999). Phenotypic characteristics of cell lines derived from disseminated cancer cells in bone marrow of patients with solid epithelial tumors: establishment of working models for human micrometastases. Cancer Res.

[B51] Baciu PC, Suleiman EA, Deryugina EI, Strongin AY (2003). Membrane type-1 matrix metalloproteinase (MT1-MMP) processing of pro-alphav integrin regulates cross-talk between alphavbeta3 and alpha2beta1 integrins in breast carcinoma cells. Exp Cell Res.

[B52] Deryugina EI, Ratnikov BI, Strongin AY (2003). Prinomastat, a hydroxamate inhibitor of matrix metalloproteinases, has a complex effect on migration of breast carcinoma cells. Int J Cancer.

[B53] Deryugina EI, Ratnikov BI, Postnova TI, Rozanov DV, Strongin AY (2002). Processing of integrin alpha(v) subunit by membrane type 1 matrix metalloproteinase stimulates migration of breast carcinoma cells on vitronectin and enhances tyrosine phosphorylation of focal adhesion kinase. J Biol Chem.

[B54] David Roodman G (2003). Role of stromal-derived cytokines and growth factors in bone metastasis. Cancer.

[B55] Muller A, Homey B, Soto H, Ge N, Catron D, Buchanan ME, McClanahan T, Murphy E, Yuan W, Wagner SN (2001). Involvement of chemokine receptors in breast cancer metastasis. Nature.

[B56] Doerr ME, Jones JI (1996). The roles of integrins and extracellular matrix proteins in the insulin-like growth factor I-stimulated chemotaxis of human breast cancer cells. J Biol Chem.

[B57] Borges E, Jan Y, Ruoslahti E (2000). Platelet-derived growth factor receptor beta and vascular endothelial growth factor receptor 2 bind to the beta 3 integrin through its extracellular domain. J Biol Chem.

[B58] Schneller M, Vuori K, Ruoslahti E (1997). Alphavbeta3 integrin associates with activated insulin and PDGFbeta receptors and potentiates the biological activity of PDGF. EMBO J.

[B59] Woodard AS, Garcia-Cardena G, Leong M, Madri JA, Sessa WC, Languino LR (1998). The synergistic activity of alphavbeta3 integrin and PDGF receptor increases cell migration. J Cell Sci.

[B60] Lev DC, Kim SJ, Onn A, Stone V, Nam DH, Yazici S, Fidler IJ, Price JE (2005). Inhibition of platelet-derived growth factor receptor signaling restricts the growth of human breast cancer in the bone of nude mice. Clin Cancer Res.

[B61] Boucharaba A, Serre CM, Gres S, Saulnier-Blache JS, Bordet JC, Guglielmi J, Clezardin P, Peyruchaud O (2004). Platelet-derived lysophosphatidic acid supports the progression of osteolytic bone metastases in breast cancer. J Clin Invest.

